# Holding Back the Tears: Individual Differences in Adult Crying Proneness Reflect Attachment Orientation and Attitudes to Crying

**DOI:** 10.3389/fpsyg.2016.01003

**Published:** 2016-07-06

**Authors:** Abigail Millings, Erica G. Hepper, Claire M. Hart, Louise Swift, Angela C. Rowe

**Affiliations:** ^1^Department of Psychology, University of SheffieldSheffield, UK; ^2^School of Psychology, University of SurreyGuildford, UK; ^3^School of Psychology University of SouthamptonSouthampton, UK; ^4^Norwich Medical School, University of East Anglia, Norwich Research ParkNorwich, UK; ^5^School of Experimental Psychology, University of BristolBristol, UK

**Keywords:** attachment anxiety, attachment avoidance, crying, emotion regulation, attitudes

## Abstract

Despite being a universal human attachment behavior, little is known about individual differences in crying. To facilitate such examination we first recommend shortened versions of the attitudes and proneness sections of the Adult Crying Inventory using two independent samples. Importantly, we examine attachment orientation differences in crying proneness and test the mediating role of attitudes toward crying in this relationship. Participants (Sample 1 *N* = 623, Sample 2 *N* = 781), completed online measures of adult attachment dimensions (avoidance and anxiety), attitudes toward crying, and crying proneness. Exploratory factor analyses in Sample 1 revealed four factors for crying attitudes: crying helps one feel better; crying is healthy; hatred of crying; and crying is controllable; and three factors for crying proneness: threat to self; sadness; and joy. Confirmatory factor analyses in Sample 2 replicated these structures. Theoretically and statistically justified short forms of each scale were created. Multiple mediation analyses revealed similar patterns of results across the two samples, with the attitudes “crying is healthy” and “crying is controllable” consistently mediating the positive links between attachment anxiety and crying proneness, and the negative links between attachment avoidance and crying proneness. Results are discussed in relation to attachment and emotion regulation literature.

## Introduction

Crying, defined as tearing for emotional reasons, is unique to humans and universal (Vingerhoets and Cornelius, 2001). Crying can be hard to control, difficult to falsify, and is imbued with an intensity unparalleled by other forms of emotional expression (Vingerhoets and Cornelius, 2001), providing a unique window into how people experience, regulate, and communicate emotions. Crying is displayed from birth into adulthood. Adults cry with varying frequency and for a variety of reasons (Vingerhoets et al., 2001a) and substantial variation is observed in proneness to crying (Vingerhoets et al., 2001b). Crying is a primary attachment behavior and attachment orientations (beliefs and strategies concerning emotions and interpersonal relationships; Bowlby, 1969; Mikulincer and Shaver, 2007) are fundamental for understanding crying. Evidence examining crying from an attachment perspective (Laan et al., 2012), however, is sparse and the underlying mechanisms of this relationship poorly understood. We explore attachment orientation differences in adult crying proneness, and the mediating role of attitudes toward crying.

### Crying in adulthood

Adult crying reflects both positive and negative emotions (Vingerhoets et al., 2001a). Bindra (1972) found that causes of crying often related to feelings of elation, dejection, or anguish. Similarly, Kottler (1996) identified physiological responses, redemption, connection to others, grief and loss, despair, joyful and aesthetic transcendence, anger and frustration, and manipulation of others, whereas Scheirs and Sijtsma (2001) identified distress, sadness, and joy (see Vingerhoets et al., 2001a for a review). Crying has intrapersonal and interpersonal functions. Theorists have argued that crying may be of intrapersonal therapeutic utility by facilitating emotional processing and acceptance of loss (Nelson, 2005; Hendriks et al., 2008). Interpersonally, crying is a key attachment behavior, intended to elicit care and comfort from close others throughout life (Bowlby, 1969; Nelson, 2005). Hendriks et al. (2008) argue that the social support elicited by crying fully explains its benefits. Empirical evidence on the outcomes of crying (including its benefits), however, is scarce. Moreover, where they exist, findings are mixed (Cornelius, 1997; for a review see Hendriks et al., 2008). Nevertheless, the belief that crying is healthy or beneficial is widespread (Cornelius, 1997). The functions of crying are moderated by individual differences. There is notable variation in the frequency with which adults report crying in everyday life (Hastrup et al., 2001). As well as established gender differences in crying behaviors (females cry more often; Frey, 1985) crying proneness has been negatively associated with both alexithymia and “distancing” coping strategies, and positively associated with neuroticism (Vingerhoets et al., 1993). But the source of this variation is not well understood.

### Adult attachment and crying

Attachment theory holds that humans are innately predisposed to form and maintain close emotional bonds with select others throughout life (Bowlby, 1969). Individual differences in adult attachment are conceptualized along the dimensions of avoidance of intimacy (i.e., a deactivating strategy), and anxiety about abandonment (i.e., a hyperactivating strategy). Those high in attachment avoidance respond to attachment-related negative affect by downplaying its importance, suppressing emotional responses, and orienting away from caregivers (Mikulincer and Shaver, 2007). Those high in attachment anxiety hyperactivate and express negative affect in an attempt to seek proximity to caregivers (Mikulincer and Shaver, 2007). Attachment security, which involves comfort with emotional expression, and the ability to regulate negative affect using internal resources, is represented by low scores on both dimensions (Brennan et al., 1998; Mikulincer and Shaver, 2007). Early work on adult attachment categorized individuals into attachment styles based on combinations of high vs. low avoidance and anxiety (Bartholomew and Horowitz, 1991).

Attachment behaviors, such as crying, ensure that humans remain in close physical proximity to their attachment figures when threat is experienced (Bowlby, 1969; Ainsworth et al., 1978; Cassidy and Shaver, 1999). In infancy and childhood, threat detection mechanisms are sensitive, thus crying behaviors are common (Zeifman, 2001). In adulthood, thresholds for crying are much higher but crying can still be evoked by attachment threat such as prolonged or unexpected separation (Zeifman, 2001; Nelson, 2005). Adults may cry to gain care from others directly (especially attachment figures). However, crying in solitude can also be conceived as seeking support from internalized representations of caregivers, which serves the same soothing and down-regulation functions (Nelson, 2005).

Preliminary evidence supports the existence of theoretically sensible attachment differences in adult crying behavior. Using single-item measures of both attachment and crying, Bartholomew and Horowitz (1991) found that those with a high-avoidant style reported lower crying frequency than those with low-avoidant style. Additionally, those with a high-anxious style reported the most frequent crying and the greatest tendency to cry in front of others (vs. alone). Using the same categorical attachment measure, Laan et al. (2012) examined self-reported crying proneness and crying in response to hearing attachment-themed songs and found that those with a high-avoidant attachment style reported the lowest proneness to crying, and the least intense crying in response to music. Those with high anxious styles reported crying more for negative reasons and less for positive reasons, and more intensely in response to music, than those with a secure style. Finally, Denckla et al. (2014) examined the relationship between attachment dimensions and vicarious crying proneness (i.e., propensity to cry when experiencing events through books or films) and found that attachment anxiety was positively related to vicarious crying for reasons associated with attachment, society, sentimentality, and compassion. Attachment avoidance was negatively related to vicarious crying concerning themes of attachment but positively related to society and sentimentality crying. By focussing on vicarious crying proneness, however, the cited research did not include real-life and personal experiences, the most common everyday triggers of crying (Bindra, 1972).

To date there has been no systematic examination of attachment differences in the full range of crying contexts, using reliable dimensional measures. Moreover, the mechanisms underlying attachment differences in adult crying have not been examined. There are two main reasons that attitudes toward crying should be considered as mechanisms. Firstly, an individual's attachment orientation is manifested behaviourally via the accessibility of cognitive schemas, containing attitudes, and beliefs relating to emotions and interpersonal relationships (Collins and Read, 1994). The two dimensions of attachment insecurity (avoidance and anxiety) have specific attitudinal components related to the expression of emotions, whereby avoidance is associated with a preference not to reveal one's true feelings or acknowledge those of others, and anxiety is associated with a desire for the emotional intimacy that results from disclosure (Brennan et al., 1998). Secondly, outcomes related to emotional expression are known to be influenced by attitudes, for example, in restricted emotionality in men (Wong et al., 2006), eating psychopathology (Meyer et al., 2010), and psychological distress in a high stress context (Brown and Grover, 1998). It is also known that attitudes toward emotional expression partially mediate the link between social anxiety and the avoidance of emotional expression (Spokas et al., 2009). Given the attitudinal components of cognitive representations of attachment, and the tendency for attitudes to predict outcomes related to emotional expression, we sought to examine attitudes toward crying as potential mediators of the link between attachment orientation and crying proneness.

### The current research

In two cross-sectional samples, we examine the relationship between attachment orientation, attitudes toward crying, and crying proneness. The motivations for our work are threefold. Firstly, we sought to examine the factor structure of the proneness and attitudes components of the most commonly-used measure of crying—the Adult Crying Inventory (ACI, Vingerhoets, 2001). While some measurement papers exist for the proneness scale (Scheirs and Sijtsma, 2001; Laan et al., 2012), to the best of our knowledge, no published works have examined the factor structure of the attitudes scale. This would provide the basis for us to examine our hypotheses in a robust way. Moreover, given that these scales are long (i.e., 55 and 24 items respectively), we hoped to identify theoretically and statistically derived short forms to facilitate future crying research.

Secondly, we sought to examine how the dimensions of attachment avoidance and anxiety predict crying proneness. We did so both by assessing self-reported crying proneness (Samples 1 and 2) and also by asking respondents about their last experience of crying in terms of recency, intensity, and duration (Sample 2). Reporting a more recent (vs. more distal) experience provides a more implicit index of crying proneness. In line with past research that used categorical attachment measures or limited crying measures (Bartholomew and Horowitz, 1991; Laan et al., 2012; Denckla et al., 2014), we predicted that (H1a) attachment avoidance would negatively predict crying proneness, and (H1b) attachment anxiety would positively predict crying proneness. Similarly, in terms of most recent episode we hypothesized that (H2a) avoidance would negatively predict crying recency, duration, and intensity, and (H2b) anxiety would positively predict crying recency, duration, and intensity.

Finally, we sought to examine for the first time the role of attitudes toward crying. Based on the above rationale concerning the role of attitudes in attachment cognitive representations and as modulators of behavior, we predicted that (H3) attitudes toward crying would mediate the relationships between attachment avoidance and anxiety, and reported crying behavior (i.e., proneness and most recent episode). We defer discussion of specific attitude dimensions until clarification of their factor structure (see below).

## Methods

### Participants and procedure

Ethical approval was received from the University of Bristol Ethics Committee (Sample 1) and the University of Southampton Ethics Committee (Sample 2) prior to commencing recruitment. For Sample 1 (*N* = 623), respondents were recruited via student mailing lists in three UK HE institutions. Personal contacts of the researchers also electronically distributed links to the study among non-student groups. Of the sample, 336 were female, 223 were male, and 64 did not disclose their gender. Participants were aged 18–81 (*M* = 23.00, *SD* = 7.10). There were missing nationality and ethnicity data (due to system error), but of the total sample, 54.4% were British (45.6% missing). Of the total sample, 53% were White, 1.4% were Black, and 3.7% were Asian (42.4% missing). Participants took part in exchange for prize draw entry for Amazon vouchers. Missing data on the variables of interest meant that mediation analyses were undertaken with *N* = 527, or *N* = 526 for analyses where Threat to Self was the dependent variable.

For Sample 2, participants (*N* = 781) were 256 undergraduates at the University of Southampton, who received course credit, and 525 volunteers who accessed the study via the internet (e.g., www.socialpsychology.org). They were invited to a study on “personality and expressing emotions” and completed the measures below online in one session among other unrelated measures. The sample comprised 572 women and 208 men (1 undisclosed gender) aged 16–65 (*M* = 23.51, *SD* = 8.46). Most were from the USA (46%) or UK (42%), and reported being of Caucasian (76%), Asian (6%), or Black (5%) ethnic origin.

### Measures

#### Sample 1

Questionnaires included measures of attachment orientation, attitudes toward crying, crying proneness, and a brief demographics section. Attachment and crying measures were counterbalanced to eliminate any priming effects from one measure to the other.

Attachment orientation was measured using the Experiences in Close Relationships scale (ECR; Brennan et al., 1998), adapted for dispositional, rather than romantic attachment (e.g., Rowe and Carnelley, 2003). The ECR is the most widely used measure of adult attachment and comprises two 18-item dimensions, tapping attachment avoidance (e.g., “*I prefer not to show people close to me how I feel deep down.”*) and attachment anxiety (e.g., “*I worry a lot about my relationships.”*). Items were rated from 1 (*strongly disagree*) to 7 (*strongly agree*). Cronbach's alphas were 0.91 for avoidance and 0.92 for anxiety, which is consistent with previous research (Brennan et al., 1998).

Participants completed the Adult Crying Inventory (ACI; Vingerhoets, 2001, cited in Vingerhoets and Cornelius, 2001). The 24-item section on attitudes toward crying covers a range of positive and negative attitudes (see Table 1 for items, 1 = *strongly disagree* 7 = *strongly agree*). Given that there is no published factor structure for this scale, we used factor analysis to derive subscales.

**Table 1:** **Exploratory factor analysis loadings for attitudes towards crying (Sample 1)**.

**Items**	**Factor**			
	**1 Feel Better**	**2 Healthy**	**3 Hatred**	**4 Control**
**10. I feel relaxed after a good cry**	**0.830**	0.007	–0.027	0.035
**12. After a good crying spell I am more optimistic about the future**	**0.802**	–0.058	0.014	0.084
15. I feel peaceful after a good cry	0.791	0.087	0.042	–0.190
**9. I find that I feel better after a good cry**	**0.779**	0.013	–0.077	0.085
11. After a good crying spell I am able to cope with my problems	0.752	–0.004	0.034	0.171
14. After crying I feel warm all over	0.576	0.201	0.175	–0.143
**8. If I let myself cry deeply, I sleep better**	**0.412**	0.125	0.063	0.333
**5. Crying is an important and effective way of dealing with life's difficulties**	–0.084	**0.979**	0.003	–0.159
**3. My life would be better if I were able to really have a good cry**	0.092	**0.728**	0.041	–0.111
**16. Crying is the healthiest thing you can do when you are feeling sad**	0.105	**0.728**	0.009	–0.157
**4. I use crying to help me feel better, when I have problems**	0.069	**0.631**	–0.038	0.159
2. I believe that it is useful to cry when life becomes stressful	0.132	0.597	–0.020	0.129
1. Crying helps me to deal with my problems	0.097	0.502	–0.095	0.179
21. I like to cry	0.076	0.432	–0.326	0.013
6. I would rather cry about a problem than keep all my sadness inside	0.044	0.403	–0.201	0.284
17. When I am not able to cry in a stressful situation I stay feeling tense	0.162	0.367	0.133	0.251
**23. I hate to cry**	0.006	–0.094	**0.734**	–0.039
**19. I feel ashamed when I am crying**	0.061	0.033	**0.723**	0.141
**13. I try not to cry when I am upset**	0.192	–0.145	**0.578**	–0.136
20. After crying I feel often more miserable than before	–0.468	0.215	0.529	0.328
**7. Under certain conditions, when things are bad, I have cried almost uncontrollably**	0.015	–0.139	0.056	**0.824**
**18. Mostly I can control my tears**	0.170	0.164	0.287	–**0.542**
24. I can manipulate others with my tears	–0.021	0.006	0.051	0.313
22. Other people generally become gentler when I cry	0.077	0.031	0.175	0.289

Items in bold comprise our recommended short form subscales. Item 7 is reversed in the short form.

The ACI proneness section asks the respondent to indicate how often they cry in each of 55 situations (see Table 2 for items; 1 = *never*, 7 = *always*). The scoring of this measure in past research has been inconsistent. For example, Laan et al. (2012) proposed two dimensions reflecting positively and negatively valenced crying proneness. Furthermore, Scheirs and Sijtsma (2001) derived a three factor solution (using factor analysis, see Table 2) depicting crying through distress, sadness, and joy, but also acknowledging one and two factor solutions. Due to the multiple published solutions, and the novelty of using this measure with UK samples, we conducted our own factor analysis and compared it against Scheirs and Sijtsma (2001).

**Table 2:** **Exploratory factor analysis loadings for crying proneness (Sample 1) compared to Scheirs and Sijtsma's (2001) study**.

**Items**	**Sample 1 FA**			**S & S 2001**
	**Threat to Self**	**Joy**	**Sadness**	
**24. …Having been humiliated/insulted**	**0.926**	–0.039	–0.101	F1
**35. …When things don't go as I want them to**	**0.875**	–0.076	–0.034	F1
41. …When I am in a blind-alley situation	0.817	0.022	–0.011	F1
**34. …When feeling self pity**	**0.801**	–0.049	–0.063	F1
23. …When I feel powerless	0.790	0.135	–0.178	F1
32. …When someone criticizes or lectures me	0.775	–0.043	0.004	F1
28. …When I experience opposition from someone else	0.769	0.149	–0.173	F1
**36. …When I feel guilty**	**0.757**	0.036	–0.050	F1
**40. …when I feel rejected by others**	**0.736**	–0.066	0.149	F1
9. …when I do not succeed in getting things together	0.726	0.033	0.066	F1
**39. …when I am in despair**	**0.716**	0.013	0.109	F1
12. …when things do not go well with work/studies	0.685	–0.012	0.121	F1
29. …when I feel frightened	0.662	–0.051	0.136	F1
5. …when I feel ashamed	0.657	0.026	0.027	F1
30. …when I feel angry	0.651	0.017	0.051	F1
19. …when involved in quarrels/conflicts	0.650	–0.074	0.190	F1
10. …when I experience disgust or contempt for something/one	0.550	0.237	–0.198	F1
49. …when I realise my own vulnerability/mortality	0.479	0.177	0.067	F1
44. …when I am ill	0.457	–0.047	0.277	F1
6. …deliberately to make someone feel sorry for me	0.416	0.053	–0.012	F1
37. …out of pity for others	0.298	0.278	0.179	F2
27. …in response to beauty of arts	0.004	0.766	–0.106	F3
**54. …when watching/hearing an admired person**	0.005	**0.719**	0.002	F3
33. …when watching awards ceremony at sporting event	–0.134	0.656	–0.038	F3
14. …when I hear a happy song	–0.039	0.637	0.039	F3
**3. I can be moved to tears by beauty of natural scenes**	0.001	**0.625**	0.037	F3
47. …when I hear national anthem or see national flag rise	–0.111	0.610	–0.146	F3
22. …while reading poetry	0.167	0.607	–0.183	F3
**55. …when I have achieved success**	0.124	**0.474**	0.090	F3
46. …when practicing religious activities	0.120	0.440	–0.213	F3
**15. …when someone does something very special for me/someone**	–0.030	**0.440**	0.410	F3
**18. …happy memories**	0.131	**0.439**	0.156	F3
**11. …when I feel very happy**	–0.117	**0.420**	0.339	F3
20. …at weddings	–0.036	0.418	0.337	F3
4. …when making love	0.195	0.358	–0.109	F3
52. …when I am reuniting with friends/family members	0.200	0.340	0.235	F3
25. …when reading certain books	0.137	0.335	0.259	F2
17. …because of problems of someone else	0.247	0.319	0.237	F2
7. …when feel relief	0.287	0.300	0.143	F3
43. …when talking with therapist/doctor	0.195	0.234	0.099	F1
**45. …while I watch sad film/TV**	–0.189	0.137	**0.834**	F2
**31. …when a tragic event happens**	0.236	–0.365	**0.788**	F2
26. …at funerals	0.011	–0.136	0.731	F2
**2. …when I say goodbye to loved ones**	0.190	–0.033	**0.588**	F2
**1….when I feel sad**	0.336	–0.088	**0.506**	F2
8. …over loss of love relationship	0.296	–0.217	0.496	F2
**16. …if I remember sad things that happened to me**	0.269	–0.009	**0.491**	F2
13. …film/TV happy ending	–0.230	0.445	0.482	F3
38. …when I experience physical pain	0.399	–0.194	0.460	F1
**50. …when I see others suffering**	0.181	0.225	**0.446**	F2
48. …when I experience painful memories	0.387	–0.047	0.420	F2
51. …when I attend/witness memorial meetings	0.058	0.239	0.391	F3
21. …when I hear a sad song	0.143	0.300	0.380	F2
42. Sometimes I laugh so hard I cry	–0.074	0.083	0.366	
53. …when I watch other people crying	0.312	0.152	0.362	F2

S & S, Scheirs and Sijtsma (2001). All items (except 3 and 42) are prefaced with “I cry” and some are truncated. Items in bold comprise our recommended short form subscales. Item 42 does not appear in Scheirs and Sijtsma's (2001) analysis, but does appear in the appendix presenting the inventory items, and therefore was included in our analyses.

#### Sample 2

Participants first completed the ACI as in Sample 1. Item orders within each section were randomized.

In addition, participants completed items from the ACI concerning their most recent crying episode. They gave a short description of “the most recent situation or event that made you feel tears in your eyes.” Participants then indicated how long ago the episode occurred (1 = *less than a day*, 7 = *more than a year ago*—reverse-coded), how long it lasted (1 = <*5 min*, 7 = *repeatedly recurring spells*), and how intense their crying was (1 = *just wet eyes*, 4 = *wet eyes, sobbing, howling, body movements, and vocalizations*). In all cases, higher scores reflected stronger crying tendencies.

Participants completed the Experiences in Close Relationships Scale Brief Version (Wei et al., 2007). Participants responded to the 6 avoidance items (α = 0.81) and the 6 anxiety items (α = 0.74) in randomized order (1 = *strongly disagree*, 8 = *strongly agree*).

## Results

First, we describe the data reduction analyses undertaken to establish subscales of the attitudes and proneness sections of the ACI with Sample 1. We then describe the replication of the factor structure in Sample 2.

Second, we describe multiple mediation analyses examining the extent to which the attitudes factors mediate the relationship between attachment orientation and crying proneness in Sample 1. We then describe the replication of these results in Sample 2.

Third, we describe our analyses pertaining to most recent crying episode (Sample 2).

### Exploratory factor analysis (sample 1)

#### Attitudes toward crying

We performed an exploratory factor analysis using principle axis factoring with promax rotation to allow factors to be correlated. The scree plot identified the presence of four factors, which accounted for 59.7% of the variance (Table 1). The first factor contained seven items reflecting the belief that crying helps one feel better (hereafter “Feels Better”). The second factor contained nine items and referred to the belief that crying is healthy (“Healthy”). The third factor contained four items reflecting a very negative view of crying (“Hatred”). The fourth factor contained four items and most referred to the belief that crying can vs. cannot be controlled (“Control”).

Due to the length of the ACI and the likely redundancy within subscales, we sought to derive a short-form of the scale for use in analyses. We selected items on the basis of: (i) retaining the strongest-loading items; (ii) eliminating conceptual redundancy (e.g., for Feels Better, item 15 was eliminated for being too similar to item 10); (iii) item distributions (e.g., item 14 was eliminated for eliciting majority disagreement); and (iv) cross-loadings >0.40 (e.g., item 20 was eliminated), while (v) retaining a consistent conceptual meaning (e.g., item 24 and 22 were eliminated because they referred to controlling other people, rather than one's own tears). In all, we selected 4 items from each of Feels Better and Healthy, 3 items from Hatred, and 2 items from Control, thus reducing the scale from 24 items to 13 items. Each short-form scale score consisted of a mean of the item scores. Given that two of the subscales contain only 2 or 3 items, we recommend that future research seeks to expand these scales to 4 items to maximize their reliability and conceptual coverage.

To assess the validity of the short-form scales, we correlated them with the full scales derived from the factor analysis. All four short-form scales correlated with their longer counterpart >0.80, suggesting that they adequately capture each factor (Table 3). Cronbach's alphas for the first three subscales were >0.70 (Table 3). The two items in the Control scale were positively correlated, *r* = 0.32, *p* < 0.001. Similar inter-item correlations within a longer scale would yield an adequate alpha, so additional items are particularly important for future use of this subscale. Given the conceptual importance of the construct of control (which partially mediated between social anxiety and emotional expression; Spokas et al., 2009), we opted to retain this subscale. Table 3 also shows the means and SDs for each subscale, by gender. Women scored significantly higher than men on all subscales, except for Hatred, where men scored significantly higher than women.

**Table 3:** **Sample 1 Means, SDs, and independent samples t-tests for short form attitudes and proneness subscales by gender**.

	**Cronbach's alpha**	**Men *M* (SD)**	**Women *M* (SD)**	***t***	***df***	**Effect size^a^**	**Correlation with long form (derived from our FA)**	**Correlation with long form factor (Scheirs and Sijtsma, 2001)**
**ATTITUDES**								
Feels Better	0.86	3.72 (1.49)	4.55 (1.28)	–6.69^**^	402.93	0.60	0.95^*^	n/a
Healthy	0.83	2.89 (1.26)	3.97 (1.27)	–9.78^**^	548	0.70	0.96^*^	n/a
Hatred	0.71	4.25 (1.57)	3.61 (1.33)	5.08^**^	548	–1.08	0.94^*^	n/a
Control	0.48	3.14 (1.44)	4.83 (1.29)	14.36^*^	548	–1.54	0.81^*^	n/a
**PRONENESS**								
Threat	0.91	2.02 (1.03)	3.93 (1.22)	19.92^**^	517.31	1.66	0.96^*^	F1 0.95^*^
Joy	0.83	1.83 (0.92)	2.83 (1.12)	–11.68^**^	527.25	0.97	0.93^*^	F3 0.94^*^
Sadness	0.87	2.94 (1.24)	4.77 (1.01)	–18.08^**^	397.33	1.65	0.96^*^	F2 0.96^*^

** *p < 0.001*,

* p < 0.05.

a Hedges g was used due to uneven sample sizes.

#### Crying proneness

We performed an exploratory factor analysis again using principle axis factoring with promax rotation for the crying proneness section of the ACI. The scree plot indicated the presence of three factors, which together accounted for 49.5% of the variance (Table 2). The first factor contained 21 items referring to a range of contexts concerning an affront to one's integrity or ego, and so we labeled it “Threat to Self.” The second factor contained 19 items mostly referring to happy or positive contexts (“Joy”). The third factor contained 15 items, most of which referred to sad or loss contexts (“Sadness”). As shown in Table 2, the solution was very similar to that reported by Scheirs and Sijtsma (2001). All items fell into the same factors as in Schiers and Sijstma's except for one in Threat to Self (which they labeled “Distress”), three in Joy, and three in Sadness—all of which loaded <0.50 on our factors.

To shorten the proneness section of the ACI, we selected 6 items for each subscale, thus reducing the items from 55 to 18 (see Table 2 for items). We followed the same criteria as those described for shortening the attitudes section. In addition, where it was possible to do so without risk of losing important conceptual coverage, we eliminated items that cross-loaded higher than 0.30 on another factor. There were two exceptions to this: item 15, “when someone does something very special for me/someone,” loaded most strongly onto Joy but also onto Sadness. Item 31, “when a tragic event happens,” loaded most strongly onto Sadness but also negatively onto Joy. However, we opted to retain these items due to their important construct coverage. Also, the process of reducing subscales made it less likely that these items would continue to cross-load in the short-form.

To assess the validity of the short-form scales, we correlated them with the full scales derived from factor analysis, as well as those presented by Scheirs and Sijtsma (2001). All three short-form scales correlated with both versions of their longer counterpart >0.90, suggesting that they adequately capture each factor (see Table 3). Cronbach's alphas for the short-form subscales were >0.80 and females scored significantly higher than males on all three subscales (see Table 3).

### Confirmatory factor analysis (sample 2)

#### Attitudes toward crying

We performed a confirmatory factor analysis on the short-form attitudes scale using SPSS AMOS 22. We modeled all 13 items as loading only onto their respective factors and allowed all four factors (i.e., Feels better, Healthy, Hatred, and Control) to covary freely. Due to missing data on several items we used Full Information Maximum Likelihood estimation. We evaluated model fit using a range of recommended indices (Hu and Bentler, 1999). These were χ^2^ (which tests the null hypothesis that the model does not differ significantly from the data, but is highly sensitive to sample size so rarely non-significant; Bollen, 1989); normed-χ^2^ (i.e., χ^2^ divided by *df* to reduce influence of sample size: good if ≤2; Ullman, 2001); comparative fit index (CFI: good if ≥0.95, acceptable if ≥0.90; Brown, 2006); and root-mean-square error approximation (RMSEA: good if ≤0.06, adequate if ≤0.08; Brown, 2006). When comparing fit of nested models, we used the significance level of Δχ^2^ and a critical value of ΔCFI = 0.010 compared to the reference model (Cheung and Rensvold, 2002; Byrne, 2010). When comparing the fit of non-nested models, we used the Akaike Information Criterion (AIC; smaller values indicate better fit; Akaike, 1987; Brown, 2006).

**Figure 1:**
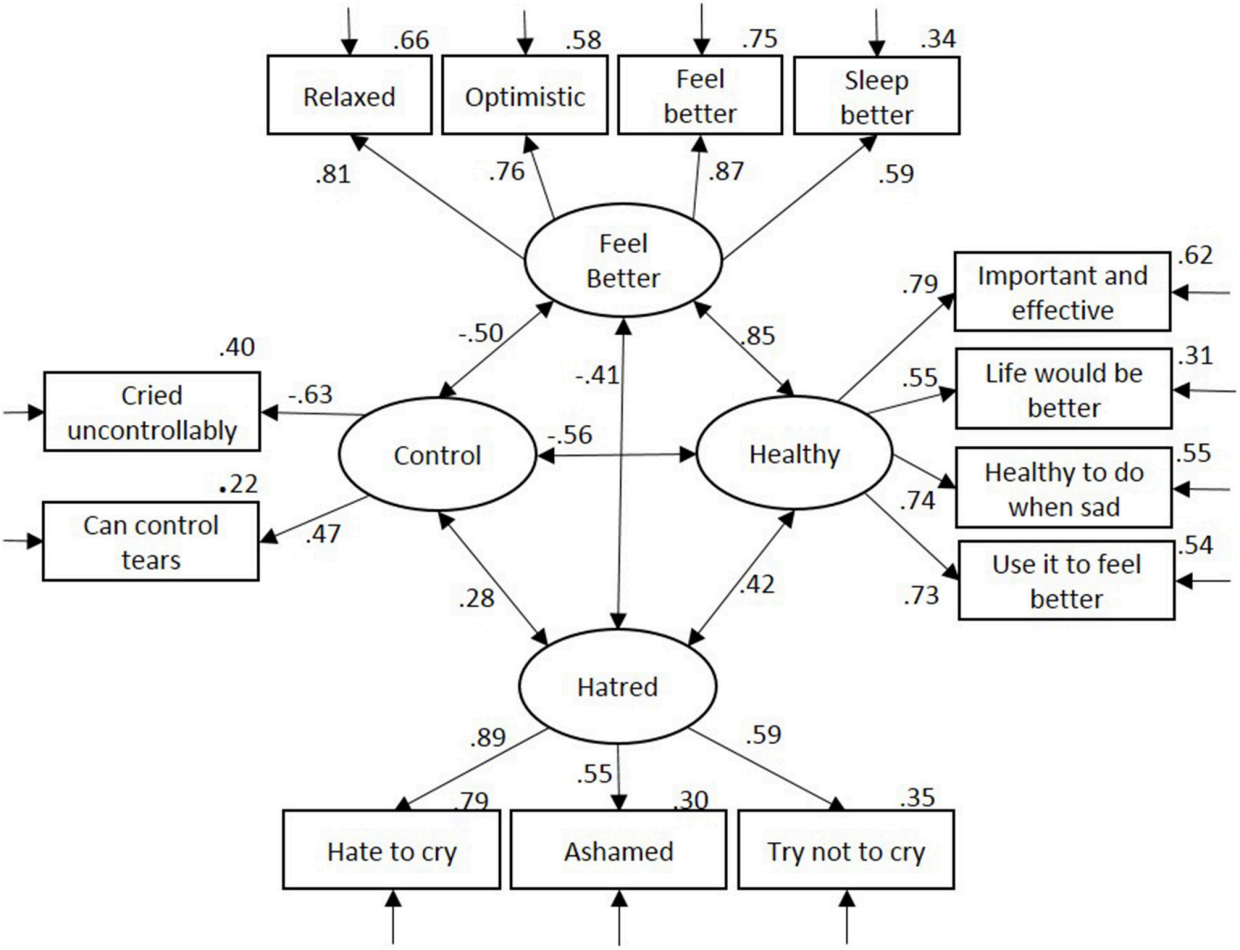
**Confirmatory factor analysis for attitudes toward crying**. Residual error terms are omitted for clarity of presentation. Variance in item explained by factor is displayed top right of each item.

The model fit was adequate and met criteria for acceptable fit, χ${}_{\left(59\right)}^{2}$ = 274.61, normed-χ^2^ = 4.65, CFI = 0.942, RMSEA = 0.068, AIC = 364.61. All items loaded significantly onto their respective factors, βs >0.467, *p*s < 0.001. As shown in Figure 1, the factors were moderately correlated, with the exception of Healthy and Feels Better, which were strongly correlated. However, an alternative model in which these two factors were combined into one showed reduced fit, Δχ${}_{\left(1\right)}^{2}$ = 87.54, *p* < 0.001, ΔCFI = 0.024, ΔAIC = 85.54^1^.

#### Crying proneness

**Figure 2:**
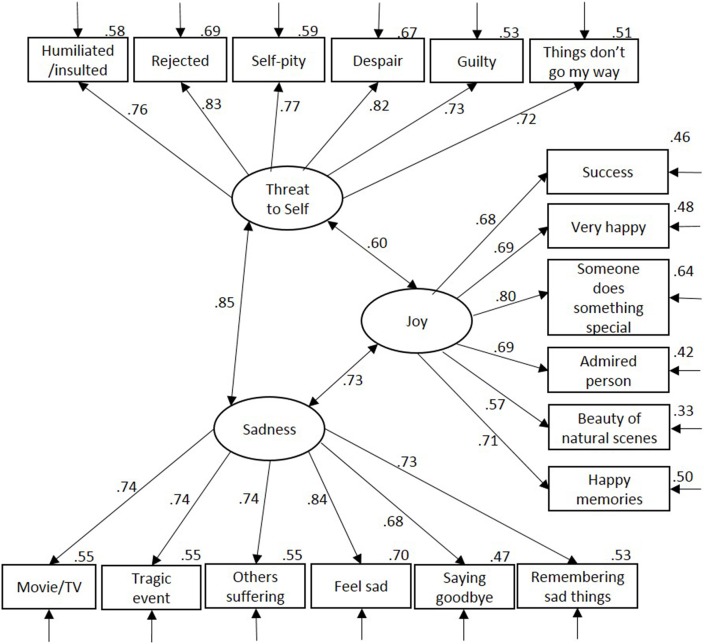
**Confirmatory factor analysis for crying proneness**. Residual error terms are omitted for clarity of presentation. Variance in item explained by factor is displayed top right of each item.

Next, we performed a confirmatory factor analysis on the crying proneness scale using the same principles as above to model the three correlated factors (i.e., Threat to Self, Sadness, and Joy). Using Full Information Maximum Likelihood estimation in the full sample, the model fit acceptably but not excellently, χ${}_{\left(132\right)}^{2}$ = 732.95, normed-χ^2^ = 5.55, CFI = 0.923, RMSEA = 0.076, AIC = 846.95. All items loaded significantly onto their respective factors, βs >0.57, *p*s < 0.001. A supplementary model in which we included only participants with complete data in order to obtain Modification Indices (*n* = 725) did not identify any substantial and theoretically sensible cross-loadings or error covariances, so we retained the model (Figure 2). The factors were moderately to highly correlated. However, an alternative model in which Threat to Self and Sadness were combined into one factor (i.e., conceptually modeling crying in positive vs. negative contexts) showed reduced fit, Δχ${}_{\left(1\right)}^{2}$ = 52.84, *p* < 0.001, ΔCFI = 0.007, ΔAIC = 50.84^2^.

### Do attitudes toward crying mediate attachment differences in crying proneness?

Having established the factor structure of both the proneness and attitudes components of the ACI, we then sought to test our hypotheses that attitudes toward crying would mediate the relationship between attachment and crying proneness. We began by examining the relationships between attachment, attitudes, and crying proneness (see Table 4). In Sample 1, attachment avoidance correlated negatively with sadness crying proneness, and correlated negatively with the attitudes that crying Feels Better and is Healthy, and positively with Hatred and Control. Attachment anxiety correlated positively with all three forms of crying proneness, and with the attitudes that crying Feels Better, is Healthy, and Hatred, but negatively with Control. In addition, all three crying proneness variables correlated positively with the attitudes that crying Feels Better and is Healthy, and negatively with Hatred and Control.

**Table 4:** **Correlations between key study variables in both samples**.

	**Attachment**		**Attitudes to Crying**				**Crying Proneness**			**Demographics**	
	**Avoidance**	**Anxiety**	**Feels Better**	**Crying is healthy**	**Hatred**	**Control**	**Threat to Self**	**Joy**	**Sadness**	**Age**	**Sex**
Attachment avoidance	−	0.20	−0.20	−0.22	0.31	0.13	−0.05	−0.01	−0.17	−0.07	−0.04
Attachment anxiety	0.08	−	0.16	0.18	0.17	−0.25	0.37	0.17	0.25	−0.18	0.16
Attitude: Feels Better	−0.13	0.21	−	0.68	−0.39	−0.31	0.37	0.31	0.42	−0.04	0.28
Attitude: Crying is healthy	−0.19	0.25	0.71	−	−0.39	−0.36	0.48	0.39	0.53	−0.01	0.39
Attitude: Hatred to cry	0.32	0.07	−0.26	−0.27	−	0.22	−0.09	−0.18	−0.23	−0.08	−0.16
Attitude: Control	0.17	−0.30	−0.32	−0.33	0.18	−	−0.56	−0.34	−0.55	0.12	−0.52
Prone: Threat to Self	−0.15	0.43	0.41	0.45	−0.12	−0.57	−	0.51	0.73	−0.21	0.63
Prone: Joy	−0.15	0.19	0.44	0.42	−0.23	−0.33	0.51	−	0.64	0.08	0.40
Prone: Sadness	−0.24	0.30	0.48	0.50	−0.23	−0.51	0.75	0.62	−	−0.05	0.63
Age (log transformed)	0.02	−0.16	−0.05	−0.05	−0.02	0.08	−0.09	0.13	−0.01	−	−0.15
Sex	−0.09	0.11	0.24	0.22	−0.11	−0.38	0.51	0.28	0.57	−0.05	−
Recent episode: Recency^a^	−0.10	0.19	0.22	0.27	−0.13	−0.27	0.44	0.33	0.41	−0.03	0.36
Recent episode: Duration^a^	0.01	0.15	0.04	0.06	0.01	−0.16	0.19	0.09	0.19	−0.10	0.16
Recent episode: Intensity^a^	−0.00	0.13	0.08	0.07	0.04	−0.18	0.14	0.05	0.13	−0.10	0.08

Sample 1 correlations appear above the diagonal, and Sample 2 correlations below the diagonal. In Sample 1, correlations ≥ ±0.09 are significant at p < 0.05, those ≥ ±0.12 are significant at p < 0.01, and those ≥ ±0.16 are significant at p < 0.001. In Sample 2, correlations ≥ ±0.08 are significant at p < 0.05, those ≥ ±0.10 are significant at p < 0.01, and those ≥ ±0.12 are significant at p < 0.001. In both studies, a positive correlation with sex indicates that women score higher than men.

a Recent episode variables in Study 2 were analyzed using Spearman's rho due to ordinal response scales.

**Figure 3:**
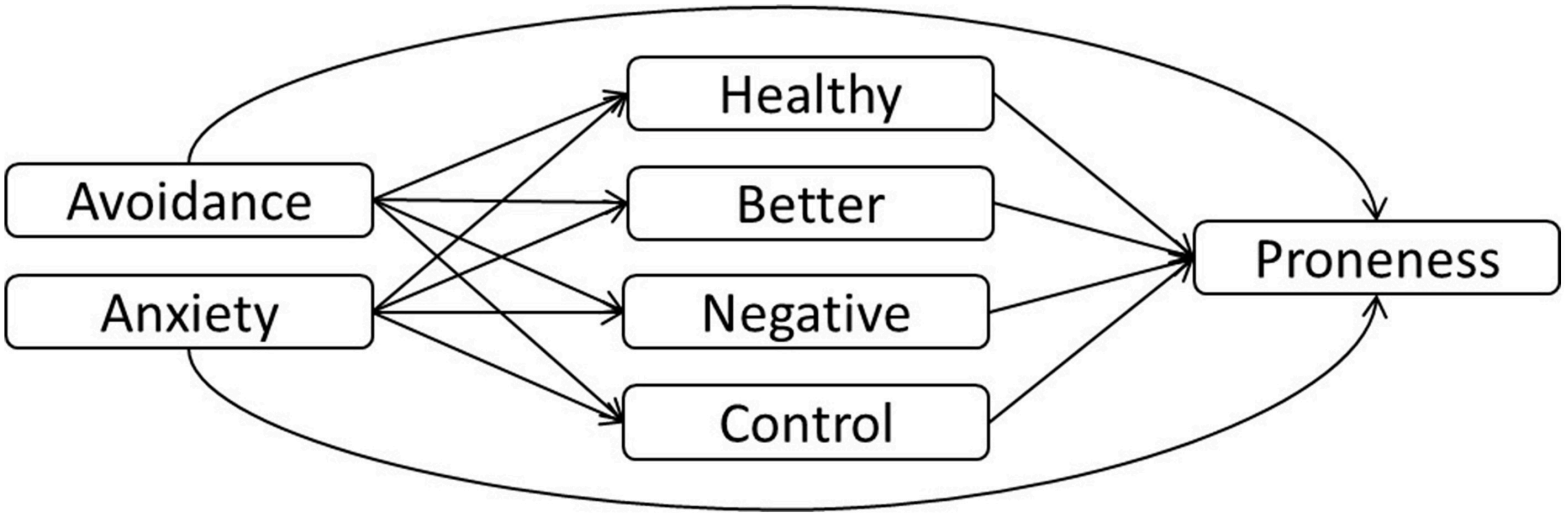
**Proposed model**.

Having broadly found support for each of the proposed pathways in our conceptual model (Figure 3), we proceeded to conduct the multiple mediation models. We first tested our model with Sample 1, and subsequently examined the extent to which we could replicate it in Sample 2, using the short form subscales in both cases. We used SPSS PROCESS (Hayes, 2013) to examine each of the three crying proneness dependent variables in turn. In each analysis, attachment avoidance (controlling for anxiety) or attachment anxiety (controlling for avoidance) was the predictor variable, and all four attitudes toward crying were entered as the mediators. In reporting results, we use terms such as “effect” and “explain” in a statistical sense only, acknowledging the correlational nature of our data.

#### Sample 1

**Figure 4:**
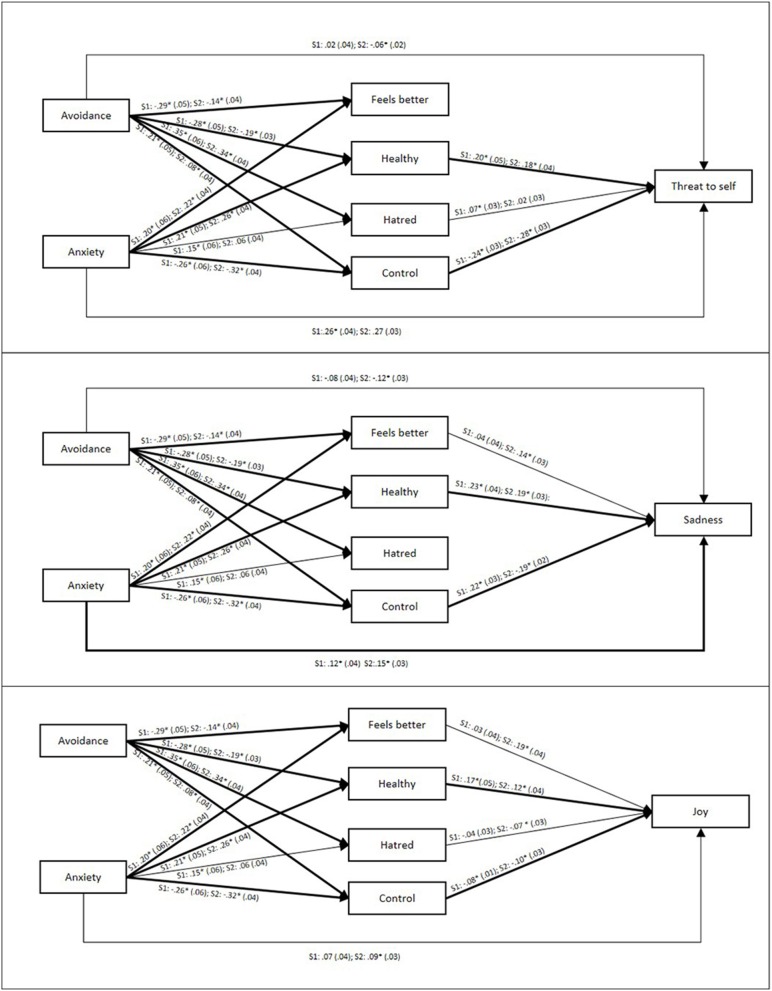
**Individual path coefficients for each relationship in the crying proneness mediation models**. S1, Sample 1, S2, Sample 2. ^*^p < 0.05. SEs are displayed in parentheses. Paths that were non-significant in both samples are omitted for ease of interpretation. Bold lines indicate that a path was significant in both samples. Coefficients shown control for age, gender, and other coefficients, including omitted non-significant ones.

In preliminary analyses, we first included gender as a moderator, allowing us to test for gender interactions in both the direct and indirect (mediated) effects. Only one significant gender moderation occurred, on the direct effect of anxiety on crying through sadness (*B* = −0.20, *p* < 0.05), indicating that attachment anxiety was a significant positive predictor of crying through sadness for men but not for women. Because this represented a single significant interaction effect out of a possible 30 (6 direct and 24 indirect), and because our primary focus was the indirect rather than main effects (which have already been established to some extent by Bartholomew and Horowitz, 1991; Laan et al., 2012; Denckla et al., 2014), in the interests of obtaining the most parsimonious models, we opted to re-run our analyses controlling for gender rather than treating it as a moderator. Direct and indirect effects are displayed in Table 5, and individual coefficients are displayed in Figure 4.

**Table 5:** **Direct and indirect effects of attachment on crying proneness via attitudes toward crying in Sample 1 and Sample 2**.

**Predictor and Criterion**		**Sample 1**			**Sample 2**		
	**Mediator (Crying Attitude)**	**B**	**(95% CI)**	***R*^2^**	**B**	**(95% CI)**	***R*^2^**
**AVOIDANCE**							
Threat to self	Feels better	–0.01	(–0.04, 0.01)	0.55	–0.01	(–0.02, 0.00)	0.54
	Healthy	–0.06	(–0.10, –0.03)^*^^a^		–0.04	(–0.06, –0.02)^*^^a^	
	Hatred	0.03	(0.00, 0.06)^*^^b^		0.01	(–0.01, 0.03)	
	Control	–0.05	(–0.09, –0.02)^*^^a^		–0.05	(–0.07, –0.03)^*^^a^	
	Direct effect	.02	(–0.07, 0.10)		–0.06	(–0.11, –0.00)^*^	
Sadness	Feels better	–0.01	(–0.04, 0.01)	0.54	–0.02	(–0.04, –0.01)^*^^a^	0.57
	Healthy	–0.07	(–0.11, –0.04)^*^^a^		–0.04	(–0.06, –0.02)^*^^a^	
	Hatred	0.00	(–0.03, 0.02)		–0.01	(–0.03, 0.00)	
	Control	–0.04	(–0.08, –0.02)^*^^a^		–0.04	(–0.06, –0.02)^*^^a^	
	Direct effect	–0.08	(–0.16, 0.00)		–0.12	(–0.17, –0.07)^*^	
Joy	Feels better	–0.01	(–0.04, 0.01)	0.28	–0.03	(–0.05, –0.01)^*^^a^	0.31
	Healthy	–0.05	(–0.09, –0.02)^*^^a^		–0.02	(–0.05, –0.01)^*^^a^	
	Hatred	–0.01	(–0.04, 0.01)		–0.02	(–0.04, –0.01)^*^^a^	
	Control	–0.02	(–0.04, –0.01)^*^^a^		–0.02	(–0.03, –0.01)^*^^a^	
	Direct effect	0.08	(0.00, 0.17)^*^		–0.04	(–0.10, 0.01)	
**ANXIETY**							
Threat to self	Feels better	0.01	(–0.01, 0.03)	0.55	0.01	(–0.00, 0.03)	0.54
	Healthy	0.04	(0.02, 0.09)^*^^a^		0.05	(0.03, 0.07)^*^^a^	
	Hatred	0.01	(0.00, 0.03)^*^^b^		0.00	(–0.01, 0.01)	
	Control	0.06	(0.03, 0.10)^*^^a^		0.09	(0.07, 0.12)^*^^b^	
	Direct effect	0.26	(0.17, 0.35)^*^		0.26	(0.21, 0.32)^*^	
Sadness	Feels better	0.01	(–0.01, 0.03)	0.54	0.03	(0.01, 0.05)^*^^a^	0.57
	Healthy	0.05	(0.02, 0.09)^*^^a^		0.05	(0.01, 0.05)^*^^a^	
	Hatred	0.00	(–0.01, 0.01)		–0.01	(–0.01, 0.00)	
	Control	0.06	(0.03, 0.09)^*^^a^		0.06	(0.04, 0.09)^*^^a^	
	Direct effect	0.12	(0.04, 0.20)^*^		0.15	(0.09, 0.21)^*^	
Joy	Feels better	0.01	(–0.01, 0.03)	0.28	0.04	(0.02, 0.07)^*^^a^	0.31
	Healthy	0.04	(0.02, 0.07)^*^^a^		0.03	(0.01, 0.06)^*^^a^	
	Hatred	–0.01	(–0.02, 0.00)		–0.00	(–0.01, 0.00)	
	Control	0.02	(0.01, 0.05)^*^^a^		0.03	(0.01, 0.05)^*^^a^	
	Direct effect	0.07	(–0.02, 0.16)		0.09	(0.03, 0.15)^*^	

* indicates a significant confidence interval (i.e., that does not include zero). Coefficients are estimates of the unstandardized B coefficients based on 1000 bootstrap resamples and controlling for age and sex. Estimates within a model that do not share a subscript differ significantly in contrast analyses. Anxiety was controlled in avoidance analyses and vice versa. All models were significant overall (ps < 0.001). Standard errors vary. Coefficients in each row indicate the indirect path through that mediator, except “direct effect” which indicates the direct effect of the attachment variable. R^2^ refers to the total model including all predictors.

Controlling for anxiety, avoidance had a significant, positive, direct effect on crying from joy. Crucially, and consistent with our hypotheses, avoidance had significant indirect (mediated) negative effects via the attitudes crying is healthy, and crying is controllable, on all three proneness factors (threat to self, sadness, and joy). That is, highly-avoidant (vs. low-avoidant) individuals' beliefs that crying is not healthy and that they can control their tears, explains their low proneness to crying. Additionally, avoidance had an indirect, positive effect on crying through threat to self via the attitude that crying is hated. The indirect effect is in the opposite direction to the zero-order correlation between hatred and crying through threat to self, implying a possible suppression pattern. Because hatred and control are correlated, when partialing out the attitude that crying is controllable, avoidant individuals' hatred of crying ironically relates to *increased* tendency to cry when feeling threatened. A similar pattern may explain the direct positive effect of avoidance on crying through joy, when controlling for the negative indirect effects described above. However, we interpret these unexpected findings with caution, especially given the notorious difficulty in replicating suppression effects (Paulhus et al., 2004).

Controlling for avoidance, anxiety had direct, positive effects on crying through threat to self and sadness. Consistent with hypotheses, anxiety also had indirect positive effects on all three proneness factors via the attitudes crying is healthy, and crying is controllable. Additionally, anxiety had an indirect, positive effect on crying through threat to self via the attitude that crying is hated. That is, highly-anxious (vs. low-anxious) individuals' beliefs that crying is healthy and that they cannot control their tears, partly explain their higher proneness to crying In addition, a suppression effect implies that, like avoidant individuals, anxious individuals' hatred of crying is associated with greater crying proneness when experiencing threat to self.

In Sample 1 we have demonstrated for the first time that the relationship between attachment and crying is mediated by attitudes toward crying. Strikingly, for both attachment dimensions, the attitudes that crying is healthy and crying is controllable were significant mediators across all three proneness factors. That is, avoidant individuals' lower proneness, and anxious individuals' higher proneness to crying, are partly explained by their relative beliefs in the healthiness and controllability of tears. Further, hatred of crying showed a suppression pattern, possibly implying that this attitude ironically related to greater proneness to crying through threat to self for insecure individuals. The attitude that crying helps one to feel better was not a significant mediator in any model. Residual direct effects for both avoidance and anxiety on crying through sadness, and anxiety on crying through threat to self, indicate that other mechanisms may also play a role in explaining attachment differences in crying. We sought to replicate these findings in Sample 2.

#### Sample 2

Similar to Sample 1, the correlations for Sample 2 (Table 4) showed that attachment avoidance correlated negatively, and attachment anxiety positively, with all three crying proneness factors. We therefore proceeded to test multiple mediation models in the same way as with Sample 1 (Table 5). Similar to our first sample, in Sample 2, the attitudes Healthy and Control consistently mediated between attachment and crying proneness in all three contexts. That is, once again individuals higher (vs. lower) in avoidance reported less proneness to crying—and this was partly explained by their beliefs that crying is unhealthy and can be controlled.

Those higher (vs. lower) in anxiety held the opposite attitudes, and this partly explained their higher proneness to crying.

However, Sample 2 also yielded some different findings from Sample 1. First, in Sample 2 we found that the attitude Feels Better significantly mediated the links between both avoidance and anxiety and crying through sadness and joy (alongside the two replicated mediators described above). Second, hatred also mediated the effect of avoidance on crying through joy, but did not mediate or suppress effects on threat to self (unlike Sample 1). Finally, Sample 2 showed more direct effects. As well as replicating the direct effects of both avoidance and anxiety on crying through sadness, and of anxiety on crying through threat to self, Sample 2 also showed a direct effect of avoidance on crying through threat to self, and anxiety on crying through joy.

#### The role of gender

Supplementary analyses using PROCESS model 8 (Hayes, 2013) examined whether gender moderated the above results in Sample 2. Significant interactions were observed for two direct effects and eight indirect effects. In the case of one direct effect (i.e., anxiety on threat to self), the coefficient was significant for both genders but stronger for women. In the case of three indirect effects (i.e., indirect effects of anxiety via Feels Better), coefficients were significant for both genders but stronger for men—in fact, all the same indirect paths were significant for men's and women's anxiety. For the remaining six effects (all concerning avoidance), the coefficient was significant for women but non-significant for men—in fact no indirect effects were significant for men's avoidance. The remaining 20 paths were not moderated by gender. Overall, attachment anxiety and its incumbent attitudes play an equal if not stronger role for men (compared to women), but attachment avoidance and its incumbent attitudes to crying appear to play a lesser role in crying proneness for men (compared to women). Sex differences in adult attachment orientations and behaviors are relatively uncommon (Feeney and Noller, 1996) but our results indicate that they exist with regards to crying (see also Frey, 1985). These findings are congruent with research showing that for men, unlike women, attachment avoidance is not a reliable predictor of support seeking behavior, of which crying is an extreme form (Simpson et al., 2002). It has been speculated that such findings may be explained by sex roles differentially affecting the link between attachment orientations and coping with stress (Shaver et al., 1996), such that attachment system activation has a higher threshold in men, may be particularly true for high avoidant men.

### Do attachment and attitudes toward crying predict most recent crying episode?

**Figure 5:**
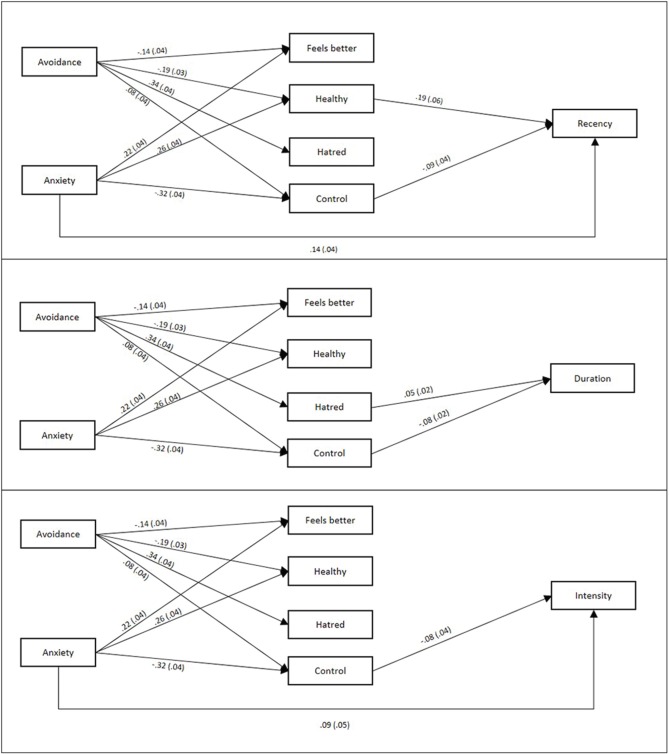
**Individual path coefficients for each relationship in the last crying episode mediation models**. SEs are displayed in parentheses. Non-significant (*p* > 0.05) paths are omitted for ease of interpretation. Coefficients shown control for age, gender, and other coefficients, including omitted non-significant ones.

In Sample 2, we additionally asked participants about their most recent crying episode. We used Spearman's rho to examine correlations with the ordinal-scale reports of participants' most recent crying episode (Table 4). Women (vs. men), and those higher (vs. lower) in attachment anxiety reported having cried more recently, for longer duration, and more intensely. Attachment avoidance correlated only with having cried less recently. Recent episode reports also correlated weakly to moderately with overall crying proneness and crying attitudes. We again used PROCESS to examine indirect effects of attachment on most recent crying episode via attitudes toward crying, controlling for age and sex^3^. Direct and indirect effects are displayed in Table 6, and individual coefficients are displayed in Figure 5.

**Table 6:** **Direct and indirect effects of attachment on most recent crying episode via attitudes toward crying (Sample 2)**.

**Criterion**	**Mediator (crying attitude)**	**Avoidance**		**Anxiety**		***R*^2^**
		**B**	**(95% CI)**	**B**	**(95% CI)**	
Recency	Feels better	0.00	(-0.01, 0.02)	–0.00	(–0.28, 0.02)	0.22
	Healthy	–0.04	(–0.07, –0. 02)^*^a	0.05	(0.02, 0.09)^*^a	
	Hatred	–0.02	(–0.05, 0.01)	–0.00	(–0.01, 0.01)	
	Control	–0.01	(–0.03, –0.003)^*^a	0.03	(0.003, 0.06)^*^a	
	Direct effect	–0.03	(–0.11, 0.05)	0.14	(0.05, 0.22)^*^	
Duration	Feels better	0.01	(–0.002, 0.03)	–0.02	(–0.04, 0.01)	0.04
	Healthy	–0.01	(0.03, 0.01)	0.01	(–0.02, 0.04)	
	Hatred	0.01	(–0.02, 0.03)	0.00	(–0.002, 0.01)	
	Control	–0.01	(–0.03, –0.001)^*^	0.03	(0.001, 0.06)^*^	
	Direct effect	0.01	(–0.07, 0.10)	0.09	(0.0001, 0.18)^*^	
Intensity	Feels better	–0.00	(–0.01, 0.003)	0.01	(–0.01, 0.02)	0.04
	Healthy	0.00	(–0.01, 0.01)	–0.00	(–0.02, 0.01)	
	Hatred	0.02	(0.005, 0.03)^*^	0.00	(–0.001, 0.01)	
	Control	–0.01	(–0.02, –0.01)^*^	0.02	(0.01, 0.04)^*^	
	Direct effect	0.00	(–0.04, 0.04)	0.03	(–0.01, 0.07)	

* indicates a significant confidence interval (i.e., that does not include zero). Coefficients are estimates of the unstandardized B coefficients based on 1000 bootstrap resamples and controlling for age and sex. Estimates within a model that do not share a subscript differ significantly in contrast analyses. Anxiety was controlled in avoidance analyses and vice versa. All models were significant overall (ps < 0.001). Standard errors vary. Coefficients in each row indicate the indirect path through that mediator, except “direct effect” which indicates the direct effect of the attachment variable. R^2^ refers to the total model including all predictors.

Overall, attachment and attitudes variables explained a statistically significant, but modest, proportion of variance in characteristics of most recent crying episode. The attitudes Healthy and Control mediated the links between both attachment dimensions and recency of crying episode. These results conceptually replicate the above-reported indirect effects for the crying proneness scale, using a more indirect and relatively implicit indicator of crying proneness. Anxiety showed direct effects on duration and intensity of crying and also indirect effects via Control. High-anxious individuals cry more intensely and are less able to stop, partly explained by their relative lack of control over crying. For avoidance, despite non-significant raw correlations, there were significant indirect effects on duration and intensity of crying via Control. High-avoidant individuals' perceived greater control related to slightly less intense and shorter crying episodes. Interestingly, avoidance also yielded a significant and positive indirect effect on intensity of crying via Hatred. Conceptually replicating the finding in Sample 1 for crying proneness, high-avoidant individuals' hatred of crying (after partialing out other attitudes) is ironically associated with their crying episodes being more intense.

Finally, supplementary analyses tested the moderating effects of sex. Significant interactions were observed for one direct effect (i.e., anxiety on recency) and four indirect effects (i.e., effects of avoidance on all three outcomes via Control, and on intensity via Hatred). In all five cases, the coefficient was significant for women but not for men—a similar pattern as observed for crying proneness outcomes in Sample 2. The remaining 25 effects, including five significant effects of anxiety and one significant effect of avoidance, were not moderated by gender.

## General discussion

In two large samples, we have identified factor structures for the proneness and attitudes components of the ACI, and recommended short forms to aid future utility. We have also delineated attitudes as a possible mechanism of the previously reported relationship between attachment orientation and crying proneness (Laan et al., 2012; Denckla et al., 2014). Our findings therefore have both theoretical and practical implications.

### Theoretical implications

We have reliably shown that the relationship between adult attachment orientation and crying proneness is partially mediated by attitudes toward crying. While there were some differences between the two sets of results, a core set of findings was replicated across both studies: the direct, positive effects of anxiety on crying through threat to self, and on crying though sadness; and indirect effects of both avoidance and anxiety on all three forms of proneness via the attitudes that crying is healthy and controllable. We discuss these in turn.

We replicate and extend the previously reported relationship between attachment and crying proneness (Bartholomew and Horowitz, 1991; Laan et al., 2012), in two samples, with the additional contribution that attitudes are an important mechanism of this relationship. Firstly, in both samples, we found evidence of a direct effect of attachment anxiety on crying due to threat to self and sadness, but not joy. Individuals with higher (vs. low) levels of attachment anxiety were more likely to cry for negative reasons. This finding is in keeping with previous literature, where those high in anxiety reported greater propensity for vicarious crying (for reasons such as attachment-related life events, society, sentimentality, and compassion, Denckla et al., 2014), and crying for negative rather than positive reasons (Laan et al., 2012). We further found that individuals with higher (vs. lower) anxiety reported crying more recently (further replicating their tendency to cry frequently) and that their most recent crying episode lasted longer. Because attachment anxiety reflects a hyperactivating affect regulation strategy (Mikulincer and Shaver, 2007), experiences and responses to threat and negative emotions are exaggerated in high anxious individuals. The proneness to crying observed in highly anxious individuals may be due to them perceiving threats more readily (Baldwin and Kay, 2003; Fraley et al., 2006) and experiencing negative emotions more keenly, leading these individuals to intensify their emotions (Mikulincer and Shaver, 2007). This intensification also accounts for the longer duration of high-anxious individuals' crying episodes, as they struggle to regulate and alleviate negative affect.

Secondly, and most importantly, we found that the attitudes crying is healthy and can be controlled mediated relationships between both attachment dimensions and all three crying proneness factors in both samples, as well as the recency of the last crying episode (in Sample 2). Attachment avoidance was negatively related to the attitude that crying is healthy, which partly explained avoidant individuals' low proneness to crying through threat to self, sadness, and joy, and less-recent crying episodes. Given that crying is an attachment behavior designed to elicit support and care from others (Nelson, 2005), avoidant individuals likely perceive it as an unwelcome emotional display and a sign of weakness that undermines their self-reliance (Cassidy, 1994). Indeed, research has found that mothers classified “dismissing-avoidant” on the adult attachment interview (George et al., unpublished manuscript) show activation of the anterior insular when presented with images of their own infants crying (Strathearn et al., 2009)—an area associated with aversive feelings such as disgust, unfairness, and pain (Montague and Lohrenz, 2007). Our finding that avoidant individuals report hatred of crying further supports this notion. Further research might examine the neural correlates of aversiveness of own crying.

Attachment avoidance was also positively related to the attitude that crying is controllable, perhaps because avoidance is essentially a deactivating affect regulation strategy. This sense of control also partly explained avoidant individuals' low proneness to crying through threat to self, sadness, and joy, and less-recent crying episodes. Avoidant individuals inhibit and suppress their emotional experience (Wei et al., 2005) and may find it (relatively) easy to avoid crying. Indeed, research has found that avoidant individuals are capable of suppressing the activation of their attachment system (Fraley and Shaver, 1997). The finding that beliefs about the controllability of crying explained their lower crying proneness is also in keeping with previous research; Spokas et al. (2009) found that attitudes regarding the importance of controlling emotional expression reduced emotional expression.

Attachment anxiety had a positive effect on the attitude that crying is healthy, which partly explained anxious individuals' relatively high proneness to crying through threat to self, sadness, and joy. Because anxious individuals, driven by unsatisfied needs for care and support, engage in hyperactivation of threat related negative emotions in a bid to seek support from caregivers (Cassidy, 1994), it follows that they would view crying positively. For high-anxious individuals crying may appear a healthy thing to do because it fits with their emotion-focused and support-seeking coping strategies (Mikulincer and Shaver, 2007).

Attachment anxiety related negatively to the attitude that crying is controllable, which also partly explained anxious individuals' proneness to crying through threat to self, sadness, and joy—as well as greater recency, duration and intensity of the most recent crying episode. Those high in attachment anxiety are emotionally reactive to external stimuli (Wei et al., 2005) and may experience spreading activation of negative affect, due to neural pathways defined by experience favoring hyperactivation rather than down-regulation. Gillath et al. (2005) found that attachment anxiety was positively associated with activation in the anterior temporal pole, which is linked with sadness, and negatively associated with activation in the orbitofrontal cortex, which is linked with emotion regulation, among participants asked to think about, and then stop thinking about negative relationship scenarios. When those high in anxiety experience sufficient cause to cry through sadness, it might be that that these same brain areas are responsible for their (in)ability to down-regulate sufficiently to control or prevent themselves from crying.

In addition to these consistent and robust findings, some effects were statistically significant in only one of our two samples. However, in all cases, the direction of the coefficients was consistent across both samples. In Sample 2 (but not in Sample 1), the attitude that crying makes one feel better significantly mediated the relationships between both avoidance and anxiety and crying through sadness and joy. That is, in both samples avoidance was negatively related to the attitude that crying makes one feel better, but only in Sample 2 did this explain additional variance in avoidant individuals' low crying through sadness and joy. Avoidant individuals have learned that there is little point in expressing negative emotions because no assistance with their downregulation is forthcoming (Cassidy, 1994). It therefore follows that they would perceive that crying would not make them feel better. Anxiety was positively related to the attitude that crying makes one feel better in both samples, and in Sample 2 this explained additional variance in anxious individuals' high crying through sadness and joy. This is likely to be linked to anxious individuals' tendency to perceive the expression of negative emotions as a means of securing access to the support of an attachment figure (Cassidy, 1994). The emergence of these additional effects in Sample 2 might reflect greater statistical power.

Additionally, hatred of crying played interesting but inconsistent roles. In Sample 1 (but not in Sample 2), significant indirect effects via hatred of crying were obtained for the links of both avoidance and anxiety with crying through threat to self. Furthermore, in Sample 2, a significant indirect effect via hatred was obtained for the link between avoidance and intensity of most recent episode. Both avoidance effects indicated a possible suppression pattern: high avoidance was associated with greater hatred of crying, which in turn was associated with a *greater* likelihood of crying through threat to self and *greater* intensity of recent crying. The conceptual replication of this finding across samples implies that it might warrant further attention. While it may be counter-intuitive that greater hatred of crying rendered avoidant individuals more likely to cry and to do so more intensely, this is likely because hatred of crying was highly correlated with the attitude that crying is controllable (which was also included in the mediation models). The positive effect of hatred of crying on crying (through threat to self) is what remains after accounting for the view that crying can be controlled. Thus, highly avoidant individuals' hatred of crying relates to a higher propensity to cry, but this is countered by their sense of control. Finally, in Sample 2, hatred of crying more straightforwardly mediated the negative association between avoidance and crying through joy. Thus, the ironic effect of high-avoidants' hatred on increased crying appears specific to episodes in which they feel threatened or are already crying—that is, when their defenses are low, perhaps because their general affect regulation strategy of deactivation fails under increased load (Mikulincer et al., 2000).

These findings have implications for interpersonal and intrapersonal functioning. Crying conveys important information to attachment figures. Avoidant individuals, who believe that crying is unhealthy but controllable, may rarely cry in front of their romantic partners in upsetting situations, giving their partners little information about their emotional state, perhaps at times at which a partner might expect to see emotions expressed. Conversely, anxious individuals, who believe that crying is healthy but uncontrollable, are likely to cry more frequently and with more ease in front of their partners, providing their partners with plenty of information about their emotional state. Such individuals may be hampered in their ability to respond to their partner crying because their own emotional reactions get in the way (Feeney and Collins, 2001). Further research should examine the interpersonal effects of different attitudes toward crying in the context of close relationships and build on work showing individual differences in implicit reactions to others' crying (Lockwood et al., 2013).

Attitudes about one's own crying also have implications for intrapersonal functioning. Crying is generally hard to control (Vingerhoets and Cornelius, 2001), and particularly so in situations of extreme stress or threat. For an individual high in attachment avoidance it may be disturbing to find that emotions can and will sometimes overtake one and result in crying. This mismatch between expectations and reality is likely to result in cognitive dissonance (Festinger, 1957) and may have negative psychological consequences.

### Practical implications

We proposed and tested short form versions of the Adult Crying Inventory's (Vingerhoets, 2001, in Vingerhoets and Cornelius, 2001) attitudes toward crying and crying proneness dimensions. We obtained a very similar factor structure for crying proneness to those reported previously (Scheirs and Sijtsma, 2001), and proposed a shortened version of this scale, reducing 55 items down to a much more user-friendly 18 items that fit the data in CFA. The subscales of the short from possess good alphas and correlate very highly with the long form, meaning that future researchers can be confident that the short form adequately captures the nuances of crying proneness covered in the long form.

We also produced a factor structure for the attitudes toward crying scale, comprising the attitudes that crying helps one feel better, crying is healthy, crying is hated, and crying can be controlled. To the best of our knowledge, this is the first time a factor structure for this aspect of the ACI has been reported. We also propose a shortened version of this scale, reducing 24 items down to 13, which fit the data in CFA. The correlations between short and long form factors were high, and the alphas for three of the four subscales were good. We therefore recommend expansion of the fourth, Control subscale, which in our short form contained only 2 items.

### Limitations and implications for future research

Despite replicating our key findings across two samples, the largest methodological limitation is the cross-sectional nature of their designs. Having highlighted attitudinal mediators in the relationship between attachment orientation and crying, further research is required to establish whether these effects are causal, both in the short term and in the longer term. This could be achieved using diary and longitudinal studies, respectively. A further limitation is the self-report nature of our work, which may result in monomethod bias, as well as demand characteristics. Future research could overcome these issues by triangulating self and close other reports, or using stimuli likely to cause crying in the lab (e.g., Cornelius, 1997).

### Conclusion

We investigated the roles of attachment orientation and attitudes toward crying, in everyday crying proneness. We found that attitudes toward crying, specifically the attitudes that crying is healthy and that crying is controllable, reliably explain attachment orientation differences in crying proneness in situations involving threat to self, sadness, and joy, as well as the strength of specific crying episodes. While high avoidant individuals reported believing that crying was both unhealthy and controllable, high anxious individuals reported believing that crying was a healthy behavior, but one that they could not control. For individuals high on either attachment dimensions, these beliefs played a pivotal role in their crying proneness and most recent crying experiences. Our results extend the literature on adult attachment and crying in theoretically meaningful and interpretable ways. We have also proposed shortened versions of both the ACI attitudes toward crying and proneness to crying scales, offering future researchers briefer tools with which to study the emotional expression process of crying.

## Author contributions

The research was designed by all the authors jointly. Sample 1 was collected by AM and AR. Sample 2 was collected by EH and CH. Statistical analyses were undertaken by AM, LS, EH, and CH. All the authors contributed to and approved the final manuscript, coordinated by AM.

## Funding

This research was supported by grants from the Economic and Social Research Council. Grant number ES/F030215/1 supported data collection and grant number ES/L001365/1 supported manuscript preparation.

### Conflict of interest statement

The authors declare that the research was conducted in the absence of any commercial or financial relationships that could be construed as a potential conflict of interest.

## Footnotes

^1^We tested whether the four-factor attitudes model fit equivalently for both women and men. First, we tested for configural invariance by fitting the model separately in each sex group. Both models fit adequately, but in the male sample one error variance estimate was negative (item 23: “I hate to cry”), rendering the solution inadmissible. Such a Heywood case can indicate insufficient indicators per latent variable (Westland, 2015) and so likely reflects the presence of only two indicators for Control. Thus, we fixed this error variance to 0.01 in the male sample analysis. The model fit adequately for both women, $\chi_{\left(59\right)}^{2}$ = 192.83, χ2/df = 3.27, CFI = 0.946, RMSEA = 0.063, and men, χ${}_{\left(60\right)}^{2}$ = 134.87, χ^2^/df = 2.25, CFI = 0.924, RMSEA = 0.078, with all factor loadings significant (βs > 0.42). Subsequently, we tested a multiple-group CFA model and imposed sequential constraints to examine between-group invariance in the measurement and structural model (Byrne, 2010). To avoid the inadmissible solution in the male sample, we constrained the relevant error variance equal across sexes (Westland, 2015). This model fit adequately, $\chi_{\left(119\right)}^{2}$ = 330.40, χ^2^/df = 2.78, CFI = 0.939, RMSEA = 0.048. To test structural invariance, we: (i) constrained item loadings to be equal across sexes, then (ii) further constrained covariances between factors to be equal (while fixing latent means to 0). The constrained model did not fit significantly worse than the unconstrained model, Δχ${}_{\left(15\right)}^{2}$ = 16.51, *p* = 0.35, ΔCFI = 0.00. Thus, the same factor structure fits the attitudes scale in women and men. Finally, we tested scalar invariance (i.e., equivalence of item intercepts), which would indicate that a person with the same underlying level of the latent factor would obtain the same score on each item regardless of their sex. We fixed the four latent means for men to 0 but allowed the means for women to vary, and constrained item intercepts to be equal. This model fit less well than the structural invariance model, ΔAIC = 54.32, implying that given the same underlying level of crying attitude, men and women report different attitudes on the scale. Inspection of individual items revealed that the intercepts were significantly different for 11/13 items (Zs ranging from |3.16–10.09|), all reflecting women's (vs. men's) greater willingness to endorse pro-crying items, and lesser willingness to endorse anti-crying items. This pattern likely reflects social desirability response bias. Nevertheless, the factor structure of the scale was equivalent across sexes.

^2^We tested for equivalence of the model across sexes in the same way as for the attitudes scale. First, we established that the model fit adequately, though no better than the overall model, in each sex group separately; women: χ${}_{\left(132\right)}^{2}$ = 548.34, normed χ^2^ = 4.15, CFI = 0.896, RMSEA = 0.074; men: χ${}_{\left(132\right)}^{2}$ = 325.74, normed χ^2^ = 2.47, CFI = 0.907, RMSEA = 0.084. Then, we followed the same multiple-group CFA procedure as for the attitudes scale to test for measurement, structural, and scalar invariance. The unconstrained model fit reasonably, χ${}_{\left(264\right)}^{2}$ = 874.37, normed χ^2^ = 2.47, CFI = 0.900, RMSEA = 0.055. Constraining the item loadings and factor covariances equal across sexes did not reduce model fit substantially, Δχ${}_{\left(18\right)}^{2}$ = 38.38, *p* = 0.003, ΔCFI = 0.004, and no individual parameters differed between sexes at *p* < 0.001. Thus, the same factor structure fits the crying proneness scale for both women and men. However, again the scalar invariance model (with equal intercepts) fit less well than the structural invariance model, ΔAIC = 70.58. Every item intercept differed between sexes (Zs = 3.05–17.85), all reflecting women's greater willingness to endorse crying proneness items. Again we note the likely influence of social desirability bias but emphasize the equivalent factor structure.

^3^Although PROCESS is not designed for use with ordinal dependent variables, we note that supplementary ordinal regressions obtained the same pattern of direct effects as those reported (but cannot test indirect effects). In addition, bootstrapping tests of indirect effects do not rely on parametric assumptions, meaning that the mediation tests are valid and lending further confidence to our conclusions.
